# The complete chloroplast genome sequence of *Pometia tomentosa*

**DOI:** 10.1080/23802359.2019.1688114

**Published:** 2019-11-12

**Authors:** Yi Wang, Xiaolong Yuan, Jinfeng Zhang

**Affiliations:** Laboratory of Forest Plant Cultivation and Utilization, Yunnan Academy of Forestry, Kunming, People’s Republic of China

**Keywords:** *Pometia tomentosa*, chloroplast, Illumina sequencing, phylogenetic analysis

## Abstract

The first complete chloroplast genome (cpDNA) sequence of *Pometia tomentosa* was determined from Illumina HiSeq pair-end sequencing data in this study. The cpDNA is 160,818 bp in length, contains a large single-copy region (LSC) of 85,666 bp and a small single-copy region (SSC) of 18,360 bp, which were separated by a pair of inverted repeat (IR) regions of 28,396 bp. The genome contains 133 genes, including 88 protein-coding genes, 8 ribosomal RNA genes, and 37 transfer RNA genes. The overall GC content of the whole genome is 37.9%, and the corresponding values of the LSC, SSC, and IR regions are 36.2%, 31.9%, and 42.4%, respectively. Further phylogenomic analysis showed that *P. tomentosa* and *Dimocarpus longan* clustered in a clade in family Sapindaceae.

*Pometia tomentosa* is the species of the genus *Pometia* within the family Sapindaceae. It is a symbol species of vallyland and a dominant uppermost canopy tree in Xishuangbanna (Southeastern Yunnan) tropical seasonal rainforest (Wen et al. 2002), and now becomes a vulnerable or rare species (Song et al. 2013). The seedling leaf of *P. tomentosa* exhibits certain shade tolerance because of its thinner leaf (Yan and Cao 2008). On sunny days in the rainy season, the leaves photosynthesis of two developing stages of young fully expanded leaves and mature leaves both existed photoinhibition (Cai et al. 2003). Therefore, *P. tomentosa* is an important plant to maintain the structure and function of tropical rainforest ecosystem (Song et al. 2006). However, there was no yet genomic report about *P. tomentosa*.

Herein, we reported and characterized the complete *P. tomentosa* plastid genome (MN106254). One *P. tomentosa* individual (specimen number: 201807019) was collected from Puwen, Yunnan Province of China (22°35′22′′N, 101°7′25′′E). The specimen is stored at Yunnan Academy of Forestry Herbarium, Kunming, China and the accession number is YAFH0012745. DNA was extracted from its fresh leaves using DNA Plantzol Reagent (Invitrogen, Carlsbad, CA).

Paired-end reads were sequenced by using Illumina HiSeq system (Illumina, San Diego, CA). In total, about 28.4 million high-quality clean reads were generated with adaptors trimmed. Aligning, assembly, and annotation were conducted by CLC de novo assembler (CLC Bio, Aarhus, Denmark), BLAST, GeSeq (Tillich et al. 2017), and GENEIOUS version 11.0.5 (Biomatters Ltd, Auckland, New Zealand). To confirm the phylogenetic position of *P. tomentosa*, other five species of family Sapindaceae from NCBI were aligned using MAFFT version 7. The Auto algorithm in the MAFFT alignment software was used to align the eight complete genome sequences and the G-INS-i algorithm was used to align the partial complex sequecnces (Katoh and Standley 2013) and maximum likelihood (ML) bootstrap analysis was conducted using RAxML (Stamatakis 2006); bootstrap probability values were calculated from 1000 replicates. *Anacardium occidentale* (KY635877) and *Mangifera indica* (KY635882) were served as the out-group.

The complete *P. tomentosa* plastid genome is a circular DNA molecule with the length of 160,818 bp, contains a large single-copy region (LSC) of 85,666 bp and a small single-copy region (SSC) of 18,360 bp, which were separated by a pair of inverted repeats (IR) regions of 28,396 bp. The overall GC content of the whole genome is 37.9%, and the corresponding values of the LSC, SSC, and IR regions are 36.2%, 31.9%, and 42.4%, respectively. The plastid genome contained 133 genes, including 88 protein-coding genes, 8 ribosomal RNA genes, and 37 transfer RNA genes. Phylogenetic analysis showed that *P. tomentosa* and *Dimocarpus longan* clustered in a unique clade in family Sapindaceae (Figure 1). The determination of the complete plastid genome sequences provided new molecular data to illuminate the Sapindaceae evolution.

**Figure 1.**
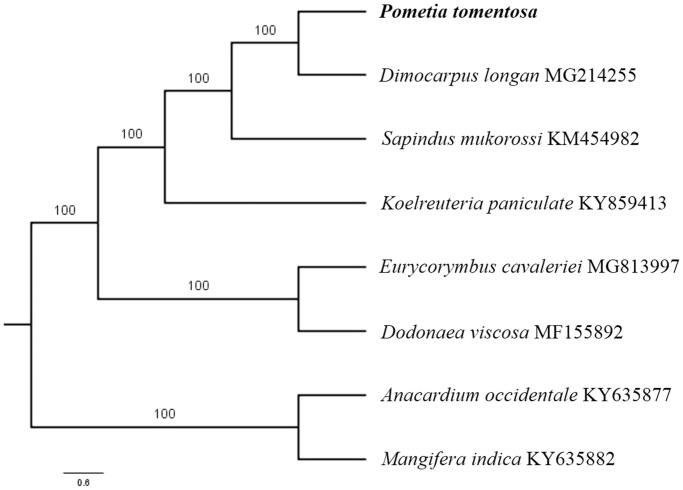
The maximum-likelihood tree based on the six chloroplast genomes of Sapindaceae family. The bootstrap value based on 1000 replicates is shown on each node.
